# Association of Biliary Source *Klebsiella pneumoniae* Pyogenic Liver Abscess With Colon Adenocarcinoma: A Case Report

**DOI:** 10.1155/crgm/8835902

**Published:** 2026-02-03

**Authors:** Dongmin Shin, Sameer Kandhi, Franklin Sosa, George Zacharia, Harish Patel

**Affiliations:** ^1^ Division of Gastroenterology, BronxCare Health System, Bronx, New York, USA, bronxcare.org; ^2^ Department of Internal Medicine, BronxCare Health System, Bronx, New York, USA, bronxcare.org

**Keywords:** colonic polyp, colorectal cancer, *Klebsiella pneumoniae*, liver abscess, pyogenic

## Abstract

**Background:**

Pyogenic liver abscesses (PLAs) commonly result from hematogenous spread or biliary tract infections, most often due to cholecystitis or cholangitis. *Klebsiella pneumoniae* is a recognized pathogen in PLA and has been associated with underlying gastrointestinal malignancies, particularly colorectal cancer. While screening for malignancy is well established in cryptogenic *K. pneumoniae* liver abscess, its role when a clear biliary source is present is less well described.

**Case Presentation:**

We report a 76‐year‐old man with a history of untreated hepatitis C and prior renal cell carcinoma who presented with right upper quadrant pain, weight loss, and leukocytosis. Imaging revealed a pericholecystic liver abscess with acute cholecystitis. The abscess was drained percutaneously, and cultures grew *K. pneumoniae*. Although imaging suggested a biliary source, colonoscopy was performed given the pathogen’s known association with colorectal neoplasia. This revealed multiple large laterally spreading tumors, including a 30‐mm ascending colon lesion confirmed as well‐differentiated invasive adenocarcinoma on biopsy.

**Conclusion:**

This case underscores the importance of considering colorectal cancer screening in patients with *K. pneumoniae* PLA, even when a biliary source is identified. Early endoscopic evaluation in such patients may facilitate timely diagnosis of occult malignancy and alter clinical management.

## 1. Introduction

Pyogenic liver abscesses (PLAs) can arise from either hematogenous dissemination of microorganisms or direct extension from a localized biliary infection [1]. Effective management requires both therapeutic drainage of the abscess and microbiological identification of the causative pathogen to guide targeted antimicrobial therapy. Determining the underlying etiology is crucial for optimizing treatment strategies; however, in approximately 50% of cases, no definitive source is identified [2].

For patients with a biliary source of infection, additional intervention may be necessary depending on the specific pathology, such as cholecystitis or choledocholithiasis, which may require a cholecystectomy or endoscopic retrograde cholangiopancreatography (ERCP) for stone removal. In cases where no biliary source is evident, a thorough evaluation for a hematogenous origin, including assessment for occult infections, endocarditis, or gastrointestinal malignancies, should be undertaken.

In patients with no identified source of infection, it is important to consider the possibility of an alternative underlying etiology, including occult malignancy such as colon cancer [3]. *Klebsiella pneumoniae* liver abscesses, in particular, have been associated with gastrointestinal malignancies, necessitating a thorough evaluation [4]. In cases where no clear biliary or hematogenous source is identified, colonoscopy should be performed as part of the diagnostic workup to rule out colorectal neoplasia.

## 2. Case Presentation

A 76‐year‐old Hispanic male with a history of hypertension, treatment‐naïve hepatitis C (Genotype 1a) with a viral load of 22 million IU/dL, and renal cell carcinoma status post left nephrectomy 28 years ago, presented with progressively worsening right‐sided abdominal pain over six weeks. His symptoms were associated with nausea, generalized weakness, and an unintentional 10‐pound weight loss. The patient had undergone a colonoscopy a decade prior, which was unremarkable, and he denied any family history of colorectal cancer.

On presentation, he was hemodynamically stable and afebrile. Physical examination was notable only for tenderness in the right upper quadrant area. Laboratory investigations revealed leukocytosis with a white blood cell count of 14.4 K/μL and anemia with a hemoglobin level of 11.8 g/dL. Liver function tests were within normal limits, including an ALT level of 7 U/L, an AST level of 11 U/L, an alpha‐fetoprotein level of 3 ng/mL, and a CA 19‐9 level of 1 U/mL. Serological testing showed hepatitis C infection, negative hepatitis B surface antigen, and positive hepatitis B core antibody. The remainder of the laboratory workup was unremarkable.

Imaging studies, including abdominal ultrasound and computed tomography (CT), demonstrated a 6.3 × 3.3 × 6.4 cm pericholecystic liver abscess (Figure 1) and acute cholecystitis, findings later confirmed by a hepatobiliary iminodiacetic acid (HIDA) scan. Additionally, mild intrahepatic biliary duct dilation was observed without common bile duct dilation, likely due to extrinsic compression from the liver abscess. Magnetic resonance cholangiopancreatography (MRCP) did not reveal any biliary stones or obstructive masses. Aspiration from the liver abscess yielded growth of *K. pneumoniae*, confirmed by culture. Antimicrobial susceptibility testing demonstrated no abnormal resistance, with the isolate being sensitive to ampicillin, aztreonam, cefazolin, imipenem, and levofloxacin. Blood and urine cultures showed no bacterial growth. The patient subsequently completed a 4‐week course of antibiotic therapy.

**Figure Figure​ 1:**
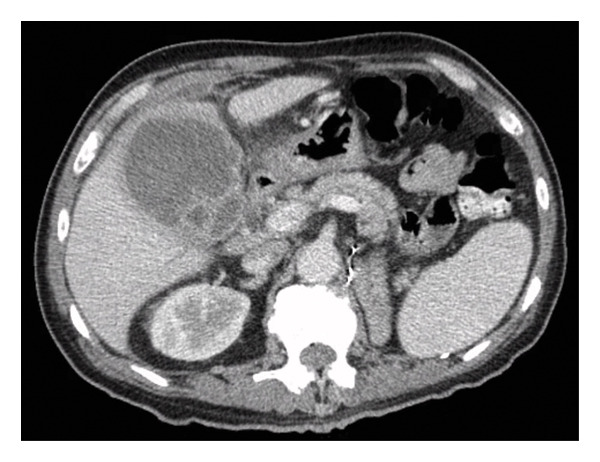
CT scan of the abdomen showing liver abscess.

**Figure 2:**
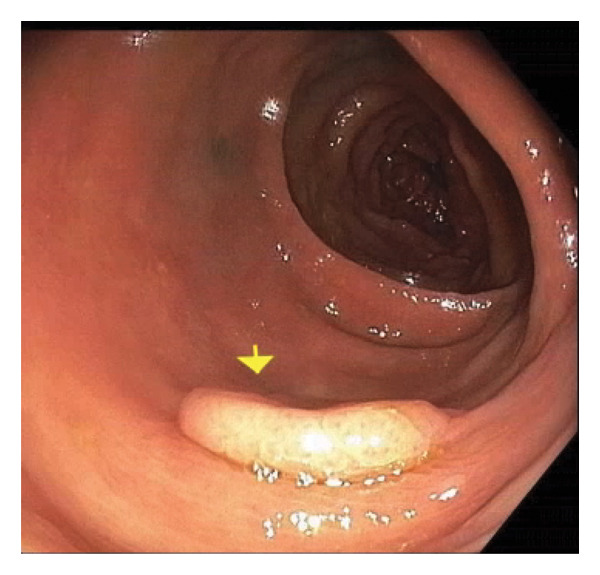
Transverse colon polyp.

**Figure 3:**
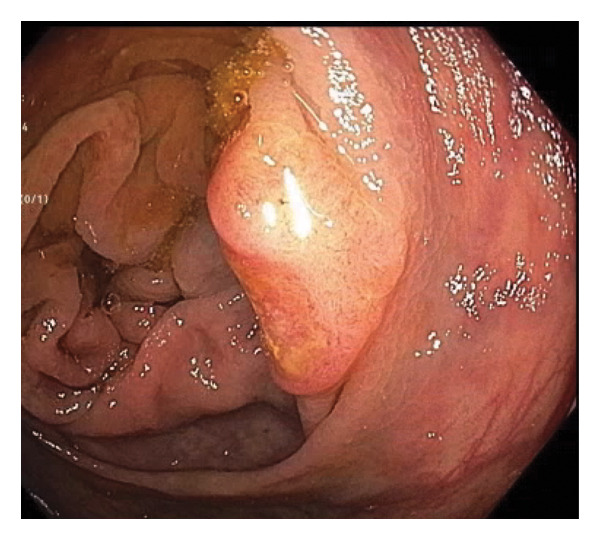
Ascending colon polyp.

Given the association between *K. pneumoniae* liver abscess and underlying gastrointestinal malignancy, a colonoscopy was performed, revealing six large carpet‐like flat colonic polyps, suspected to be laterally spreading tumors (LSTs) of varying morphologies, including granular and nongranular subtypes with flat‐elevated and pseudo‐depressed surfaces. These polyps ranged from 10 mm to 30 mm in size, extending from the ascending to transverse colon (Figures 2 and 3), with additional smaller polyps throughout the colon. Biopsy of a 30‐mm polyp in the ascending colon confirmed a well‐differentiated invasive adenocarcinoma arising in the background of tubular adenoma. Additional biopsies identified sessile serrated adenomatous polyps and tubular adenomas.

The patient was referred to surgery and oncology for further evaluation and definitive management.

## 3. Discussion

CRC can cause defects in the mucosal barrier of the gut, making it easier for bacteria to invade the portal system with hematogenous spread to the liver or enter the systemic circulation. In line with this mechanism, certain infections (e.g., *Streptococcus gallolyticus* bacteremia, anaerobic bacteremia, and PLA) have been associated with the increased incidence of CRC and can be the first sign of CRC in otherwise asymptomatic patients [3].

US population–based studies revealed that the incidence of PLA has been increasing over the past decades [4, 5]. This pattern was also observed in other countries. Jepsen et al. [6] conducted a population‐based study in Denmark and found that the incidence of PLA increased from 0.6 to 1.8 cases per 100,000 person‐years between 1977 and 2002 [6]. Similarly, in Taiwan, the estimated incidence of PLA rose from 10.8 to 15.4 cases per 100,000 person‐years between 2000 and 2011 [7].

PLA is a severe bacterial infection of the liver leading to the formation of pus‐filled cavities and is associated with significant morbidity and mortality. PLAs can develop through several routes, including the portal vein, the biliary tract, and the hepatic artery. They can also occur from direct physical trauma to the liver, spread from nearby infections, and sometimes the source of the infection may remain unknown (i.e., cryptogenic) [8].

The relationship between PLA and CRC has been a significant topic of research. A nationwide cohort study in Taiwan by Kao et al. found that PLA patients have a significantly higher risk of developing various cancers compared to the general population, including liver, biliary tract, and colorectal cancers. This increased risk persisted throughout the follow‐up period, with a notably higher risk within the first 3 months following diagnosis [9].

Studies have also demonstrated that patients with PLA, particularly those caused by *K. pneumoniae*, have a higher risk of concurrent or subsequent colorectal cancer, raising the question of the need for screening colonoscopy in such patients. Mohan et al. [10] found in a systematic review that 7.9% of cryptogenic PLA patients had CRC compared to 1.2% of controls, indicating a 7‐fold increased risk. Most colonic lesions in PLA patients were left‐sided, and 93.1% of cases were caused by *K. pneumoniae* [10]. Another review by Qu et al. [11] that showed a strong association between PLA and CRC reported that 80% of the cases were from Eastern Asian countries, with *K. pneumoniae* being the most common pathogen. Most CRC tumors were found in the sigmoid colon (40.9%) and the rectum (27.3%) [11]. A retrospective study by Huang et al. [11] consisting of 2294 patients also showed a strong association between *K. pneumoniae* infection and CRC. The study showed that the risk for CRC was 2.68 times greater for patients with *K. pneumoniae* PLA than for those with non‐*K. pneumoniae* PLA [12].

Suzuki et al. [3] conducted a patient‐level matched retrospective cohort study over 18 years across 127 hospitals, involving 8286 patients diagnosed with PLA. The study revealed that the incidence of CRC was significantly higher in patients with PLA compared to the control group in the first 3 years following a PLA diagnosis, with hazard ratios of 3.64 at 0.5 years, 2.51 at 1 year, 1.74 at 2 years, and 1.41 at 3 years. Notably, this increased risk was not observed in patients whose PLA was secondary to cholangitis or cholecystitis [3].

A retrospective study from South Korea revealed multiple colonic lesions (24.3%) in association with cryptogenic PLAs, including colon cancer, LST with high‐grade dysplasia, colonic ulcers, and polyps [13].

## 4. Conclusion

Although not included in the current screening guidelines, many studies suggest the importance of considering a colonoscopic examination to detect occult CRC in patients with cryptogenic PLA, especially those caused by *K. pneumoniae*, as it may be the first manifestation of CRC in an otherwise asymptomatic patient.

Our case also highlights that colonic lesions, including malignancy, can be found even in non–East Asian, nondiabetic patients with a likely hepatobiliary source of infection for *K. pneumoniae* PLA and the importance of a thorough complete examination including the right colon.

## Funding

No funding was received for this study.

## Consent

No written consent was obtained from the patient as the institutional protocol does not require written consent when no identifiable patient information is included in the manuscript.

## Conflicts of Interest

The authors declare no conflicts of interest.

## Data Availability

All data pertaining to this case report are included in this manuscript. Data supporting this case report are available from the corresponding author upon reasonable request.
